# Next-Generation Orthodontics: Functional Resins, Biomechanics, Biocompatibility, and Current Clinical Reality of Direct 3D-Printed Aligners

**DOI:** 10.3390/jfb17030129

**Published:** 2026-03-09

**Authors:** Yulong Zhang, Benjamin M. Wu

**Affiliations:** The ADA Forsyth Institute, Somerville, MA 02143, USA; yzhang@forsyth.org

**Keywords:** direct 3D-printed aligners, photocurable resins, functional material engineering, biomechanics, shape memory polymers, post-processing, microplastics, biocompatibility

## Abstract

The orthodontic landscape is currently witnessing a significant technological evolution with the emergence of direct 3D-printed aligners (DPAs), which promise to close the digital workflow loop by eliminating the geometric limitations and solid model waste inherent to traditional thermoformed clear aligners (TCAs). This review provides a comprehensive analysis of the material science governing this transition from inert thermoplastic sheets to reactive photocurable resins. We explore the fundamental chemistry of DPA materials, and the pivotal role of post-processing in ensuring mechanical integrity and biocompatibility. Beyond passive mechanics, this review highlights preclinical research in functional material engineering, detailing how experimental DPAs are being investigated for the integration of antibacterial agents, remineralization fillers, and drug delivery systems. Furthermore, we evaluate the limited but emerging clinical data on DPAs, contrasting their shape-memory properties and force delivery profiles with conventional appliances, while critically addressing emerging safety concerns regarding monomer elution and microplastic generation. We conclude that while DPA technology offers superior dimensional control, comprehensive life cycle assessments and long-term in vivo trials are essential to fully substantiate their clinical efficacy, overall sustainability, and potential as advanced orthodontic appliances.

## 1. Introduction: The Orthodontic Material Revolution

### 1.1. Historical Evolution

The history of orthodontics is fundamentally a history of material science evolution, where each epoch is defined by the limitations and capabilities of the materials available to the clinician. The early 20th century, often referred to as the “Era of Metals,” was dominated by the use of precious metals and later stainless steel for bands and wires. This created a “fixed” mechanics paradigm that offered precise three-dimensional control over tooth movement but suffered from poor aesthetics and significant iatrogenic potential due to plaque accumulation [1,2]. The introduction of composite bonding and ceramic brackets in the late 20th century addressed aesthetic concerns but maintained the fixed appliance workflow, merely camouflaging the machinery of force delivery [3,4].

The third revolution occurred in the late 1990s with the popularization of thermoformed clear aligners (TCAs), most notably by Align Technology [5]. This system introduced a hybrid workflow: utilizing computer-aided design (CAD) to stage tooth movements virtually, followed by computer-aided manufacturing (CAM) to print stereolithographic models for each stage. Thermoplastic sheets were then vacuum-formed over these physical models to create the appliance. While TCAs democratized aesthetic orthodontic treatment and introduced mass customization to the field, the manufacturing workflow remained fundamentally indirect. The reliance on an intermediate physical model introduced a source of error and material waste that has persisted for over two decades.

The current, fourth revolution—direct 3D-printed aligners (DPAs)—represents the closure of the digital loop. By eliminating the physical model entirely and printing the aligner shell directly from liquid resin via additive manufacturing, DPAs promise a purely digital workflow [6,7]. This shift is not merely procedural, it allows for the manipulation of the aligner’s geometry and internal structure in ways impossible with thermoforming, enabling mass customization, variable thickness profiles, and a reduced environmental footprint by eliminating large volumes of non-biodegradable model waste [8].

### 1.2. The Biomechanical Bottleneck of Thermoforming

Despite their market dominance, thermoformed aligners suffer from inherent biomechanical and material limitations that restrict their clinical efficacy and predictability. These limitations are largely artifacts of the manufacturing process itself rather than the design of the appliance.

#### 1.2.1. Geometric Limitations

The thermoforming process involves stretching a heated thermoplastic sheet over a dental model. This stretching is inherently non-uniform; the material thins significantly over prominent line angles and incisal edges, often reducing the aligner’s thickness by up to 50% in these critical areas [9]. A clinical study documenting dimensional changes found that 0.75 mm plastic sheets varied between 0.38 mm and 0.69 mm after thermoforming [7]. Since the force exerted by an orthodontic appliance is proportional to the cube of its thickness, a 10% reduction in thickness can theoretically reduce exerted forces by approximately 30% [10]. This variability renders force delivery unpredictable, as the clinician cannot control the exact thinning profile of the sheet over specific tooth anatomies. Furthermore, thermoforming is subtractive in its final stage (trimming) and constrained by the initial sheet thickness; it cannot produce complex internal geometries, undercuts that exceed the path of draw, or variable thickness profiles within a single aligner to reinforce anchorage or apply differential forces [11].

#### 1.2.2. Material Fatigue and Stress Relaxation

Thermoplastic materials, particularly polyethylene terephthalate glycol (PETG) and single-layer polyurethane (TPU), exhibit rapid stress relaxation [12]. Upon initial insertion, they exert high peak forces that decay exponentially within the first few hours due to the viscoelastic nature of the polymer. This phenomenon, known as stress relaxation, means that for the majority of the recommended wear interval (typically 1–2 weeks), the aligner may not be delivering orthodontic force levels sufficient to move the teeth. While multilayer materials (e.g., SmartTrack) were developed to mitigate this by combining rigid and elastic layers [13,14], the fundamental limitation of the thermoplastic manufacturing process—the inability to decouple stiffness from elasticity within the same material block—remains a barrier.

#### 1.2.3. Processing Defects

The thermoforming process induces internal residual stresses within the polymer chains as they are rapidly heated, stretched, and cooled. Exposure to the intraoral environment—specifically the combination of body heat (37 °C) and water absorption from saliva—can trigger the release of these residual stresses, leading to dimensional instability and warping over time [15]. Additionally, the reliance on physical models means that any inaccuracy in the 3D-printed model is transferred to the aligner’s intaglio surface, compounding errors and potentially altering the force vector.

### 1.3. The Promise of Direct 3D Printing

Direct 3D printing circumvents the limitations of traditional thermoforming by utilizing additive manufacturing to build the appliance layer-by-layer, allowing for a level of precision and customization previously unattainable, effectively shifting the focus from mass production to true mass customization. The most significant advantage of this workflow is the design freedom it affords, specifically the capacity to program site-specific thickness variations within a single aligner. Clinicians can engineer appliances with thicker cross-sections (≥0.75 mm) in posterior segments to reinforce anchorage rigidity, while simultaneously employing thinner, more compliant profiles (e.g., 0.5 mm) in the anterior region. This geometric customization creates a “variable stiffness” appliance that delivers gentle, constant forces to periodontally compromised dentition or facilitates complex rotational movements—a localized tuning of force systems that uniform thermoformed sheets cannot match [16].

Beyond design flexibility, DPAs offer superior dimensional fidelity by eliminating the cumulative errors associated with model printing, vacuum forming, and manual trimming. Research comparing DPA resins (such as Tera Harz TC-85) against premium thermoplastics (e.g., Essix ACE and Zendura FLX) demonstrates that printed aligners achieve significantly higher accuracy. Quantitative analysis using Root Mean Square (RMS) error values—where a lower number indicates higher precision—shows that DPAs exhibit deviations as low as 0.140 mm, compared to 0.188–0.209 mm for thermoformed counterparts [17]. This precision translates to an improved clinical fit and more predictable tooth movement, as the “clearance” gap between the appliance and the tooth can be controlled with micron-level accuracy to optimize the movement path [18].

Finally, the adoption of DPAs introduces the potential for a more sustainable workflow by obviating the need for disposable dental models. Traditional aligner fabrication generates substantial non-biodegradable plastic waste annually; in contrast, DPAs create a streamlined process where the primary material consumed is the aligner resin itself [16,19]. Furthermore, the elimination of the intermediate model printing and thermoforming steps significantly reduces total fabrication time and costs. This operational efficiency could ultimately facilitate “same-day aligner” delivery protocols in clinical settings, thereby enhancing both patient satisfaction and practice productivity [20]. However, while DPAs hold the promise of establishing a “green workflow” and expediting treatment, widespread clinical implementation and comprehensive life cycle assessments remain necessary to fully validate these environmental and operational advantages.

### 1.4. Methodology and Scope

This paper is presented as an expert opinion-based narrative synthesis rather than a systematic or quasi-systematic review. The primary objective is to provide a comprehensive overview of the material science, biomechanical principles, and emerging clinical realities surrounding DPAs. To capture the current state of this rapidly evolving field, we conducted a literature search across electronic databases, including PubMed/MEDLINE, and Google Scholar, focusing primarily on recent publications (since 2019). Search terms included combinations of “direct printed aligners,” “3D-printed clear aligners,” “shape memory polymers,” “photocurable orthodontic resins,” and “aligner biocompatibility.” While this review strives for a thorough evaluation of recent in vitro materials research and available pilot clinical trials, it does not employ strict systematic inclusion or exclusion criteria. Consequently, we acknowledge the inherent potential for selection bias. Furthermore, given the nascent stage of DPA technology, this review strives to carefully differentiate between experimentally substantiated data and theoretical or proof-of-concept applications, particularly concerning long-term clinical efficacy and environmental impacts.

## 2. Material Science of DPAs

The transition to DPAs represents a fundamental shift in orthodontic material science, moving beyond the capabilities of traditional thermoformed appliances. This transition is underpinned by the development of sophisticated photocurable resins. Unlike the inert, pre-polymerized thermoplastics used in TCAs, DPA materials begin as reactive liquid resins that must be polymerized in situ into a solid network [21]. These are not generic dental resins but highly specialized formulations designed to withstand the cyclic loading of mastication while delivering continuous orthodontic forces. This requires a sophisticated understanding of photopolymer chemistry to balance competing material properties.

### 2.1. Photopolymer Chemistry Fundamentals for DPA Resins

The dominant technology for DPAs is photopolymerization, where liquid monomers and oligomers are cross-linked into a solid 3D network upon exposure to UV or visible light, typically at wavelengths of 385–405 nm [21].

The resin formulation for DPAs typically consists of three primary components:

(a) Monomers and Oligomers: These are the building blocks of the polymer network. Oligomers (short polymer chains) provide the bulk properties (toughness, flexibility), while reactive diluent monomers reduce viscosity to allow printing and contribute to cross-linking density.
(b) Photoinitiators: Molecules that absorb light energy and generate reactive species (free radicals or cations) to start the polymerization chain reaction.
(c) Additives: These include UV blockers (to control cure depth and prevent “bleeding” of light), pigments, and stabilizers to prevent premature curing [22,23].

Upon exposure to light, the photoinitiator decomposes into radicals that attack the double bonds of the monomers (usually acrylates or methacrylates), linking them into long chains and cross-linked networks. The density of these cross-links determines the final mechanical properties of the aligner [22]. Unlike thermoplastics, which rely on entangled polymer chains held together by weak intermolecular forces, DPA resins form thermosets (or highly cross-linked thermoplastics) characterized by strong covalent bonds.

A critical chemical barrier for the photopolymerization in DPAs is oxygen inhibition [24,25]. Methacrylate polymerization proceeds via free-radical propagation. Atmospheric oxygen acts as a radical scavenger, reacting with propagating radicals to form stable peroxyl radicals, effectively terminating the chain reaction at the surface [25]. This results in an uncured, cytotoxic “inhibition layer” that compromises mechanical integrity and biocompatibility, necessitating specific post-processing strategies such as intense washing or UV post-curing.

Moreover, formulating these resins requires balancing three conflicting properties, often referred to as the “Trade-off Triangle”:

(a) Transparency: Essential for patient aesthetic acceptance. High crystallinity or phase separation can improve strength but often causes opacity or haze. Achieving optical clarity requires amorphous polymer structures or nano-scale phase separation that does not scatter visible light [14,26,27].
(b) Toughness: The ability to withstand insertion/removal forces and bruxism without fracture. This requires a high elongation at break. However, increasing toughness often reduces the modulus and increase stress relaxation [28].
(c) Stress relaxation: A critical material property governing the aligner’s working range. A low stress relaxation ensures the delivery of light, continuous forces necessary for efficient periodontal remodeling within the “biological window”, avoiding the rapid force decay associated with rigid materials [29,30].

### 2.2. Design Rationales: Soft vs. Hard Segments

The mechanical performance of aligner resins is fundamentally dictated by the architecture of the polymer backbone, specifically the ratio and arrangement of “hard” and “soft” segments within the polymer chain. This concept, borrowed from thermoplastic elastomer chemistry, is now being applied to photopolymers.

(a) Hard Segments: These are typically composed of urethane units, aromatic rings, or bulky cycloaliphatic groups. They provide the polymer with stiffness (modulus), physical cross-linking, and thermal stability. They are responsible for the aligner’s ability to exert force on the tooth and resist deformation under load.
(b) Soft Segments: These consist of long aliphatic chains, polyols, or polyethers. They provide flexibility, elongation, and elastic recovery. They allow the aligner to deform during insertion and removal without exceeding its yield point and fracturing.

Optimizing this ratio is critical for DPA performance. Resins like LuxCreo’s ActiveMemory™ polymer utilize a sophisticated multi-component architecture where hard segments maintain stability at body temperature (preventing the aligner from becoming too soft in the mouth), while soft segments allow for reversible deformation. This effectively decouples intraoral mechanical stability from the thermal activation required for shape recovery, addressing a key limitation of earlier shape-memory polymers [31,32].

A sophisticated method for toughening DPA resins involves polymerization-induced phase separation (PIPS). In this process, a homogeneous resin mixture separates into distinct microscopic phases (e.g., a hard matrix and soft rubbery domains) during the curing process [33]. This heterogeneous morphology is critical for toughness; the soft domains can arrest crack propagation (crazing), while the hard matrix maintains the overall shape and modulus. PIPS allows for the creation of materials that are both stiff and tough, a combination that is difficult to achieve with homogenous polymer networks [34].

### 2.3. Chemical Nature of Resins Used in DPA Resins

The efficacy and safety of DPAs are intrinsically linked to the chemical composition of the resins used in their fabrication. Several classes of resins have been explored for this application, each with unique characteristics that influence the aligner’s performance.

Polyurethane-based resins are frequently employed due to their inherent balance of clarity, strength, and flexibility, which are essential for delivering the continuous and controlled forces required for orthodontic tooth movement [23]. A prominent example is Graphy’s Tera Harz TC-85, which is composed of a polyesterurethane polymer [35]. The desirable properties of this chemical family are well-established in conventional orthodontics; for instance, Align Technology’s Smart Track material, used in their thermoformed aligners, is similarly based on a multilayer aromatic thermoplastic polyurethane/copolyester [36]. The selection of polyurethane resins often stems from their favorable mechanical properties and the potential for enhanced patient comfort [37].

Methacrylate-based resins also play a significant role in DPA manufacturing. These photopolymerizable resins, such as Formlabs’ Dental LT Clear, which comprises a methacrylate oligomer and glycol methacrylate, are known for their good biocompatibility (Class IIa biocompatible material) and ability to form strong and durable structures upon curing [38]. Within this class, various monomers like Urethane dimethacrylate (UDMA) and Triethylene glycol dimethacrylate (TEGDMA) are commonly used, often in combination with Polymethyl methacrylate (PMMA) and sometimes incorporating nanoparticles to enhance specific properties. The robust nature of cured methacrylate resins makes them suitable for applications requiring dimensional stability and resistance to intraoral forces.

Vinyl ester-urethane polymers represent another category of resins used in the direct 3D printing of aligners. Tera Harz TC-85, besides being a polyesterurethane, also falls under this classification as an aliphatic vinyl ester-polyurethane polymer functionalized with methacrylate. The incorporation of vinyl ester groups into the polyurethane backbone can enhance the resin’s photoreactivity during the 3D printing process and contribute to improved mechanical properties in the final aligner [39,40]. Building upon the distinct advantages of these individual polymer families, hybrid formulations have emerged as a highly effective chemical approach. As will be detailed in Section 2.5, contemporary material science has moved away from relying on single monomer networks, instead focusing on synthesizing these components to meet the rigorous clinical demands of DPA therapy.

### 2.4. Under-Development Chemistries

#### 2.4.1. Non-Isocyanate Polyurethanes (NIPUs)

Conventional polyurethanes utilize isocyanates, which are toxic, moisture-sensitive, and pose health risks during handling and elution. NIPUs are emerging as a “greener” and safer alternative, synthesized via the reaction of cyclic carbonates with polyamines [41]. These materials eliminate the risk of isocyanate residues and offer highly tunable mechanical properties via thiol–ene click chemistry. Recent research demonstrates that NIPU elastomers can be 3D printed using rapid projection photopolymerization and exhibit excellent biocompatibility, attributed to their “green” synthesis from bio-derived diamines (such as cadaverine) [42]. These materials display remarkable mechanical tunability, where stiffness, flexibility, and elasticity can be precisely controlled by adjusting the ratios of branched thiol crosslinkers (e.g., tetra- vs. linear thiols). The ability to spatially pattern these distinct mechanical properties into complex, compliant structures—coupled with verified low cytotoxicity—makes them a promising candidate for future, safer aligner materials.

#### 2.4.2. Hot Lithography and High-Viscosity Resins

Standard SLA/DLP relies on low-viscosity resins to allow for rapid recoating of layers. However, low-viscosity monomers (diluents) often produce brittle polymers with low impact strength due to their short chain lengths and high cross-link density [43]. Hot lithography processes address this by heating the resin vat up to 120 °C, allowing the use of high-viscosity resins to overcome the mechanical weakness of DPAs. This thermal energy lowers the viscosity of high-molecular-weight, long-chain oligomers to a printable range. This allows the use of resins that are extremely tough and impact-resistant at room temperature, mimicking the properties of engineering thermoplastics like ABS or Polypropylene. This technology is promising for producing aligners that do not fracture under high occlusal loading while maintaining the necessary elasticity for tooth movement [44].

While high-viscosity hot lithography offers a pathway to mechanically superior aligners (tougher, less brittle), it currently struggles with dimensional inaccuracy due to thermal shrinkage, slower production speeds, and increased hardware complexity [45,46,47]. Balancing the need for heat (to lower viscosity) with the risk of warping the aligner remains a critical engineering hurdle.

#### 2.4.3. Epoxy/Hybrid Resins and Cationic Curing

While the majority of commercial 3D-printed aligners utilize free-radical polymerization of acrylates or methacrylates, cationic curing systems based on epoxies and oxetanes offer a distinct chemical alternative. Unlike free-radical systems, which are significantly inhibited by atmospheric oxygen and often result in an under-cured surface layer known as the “oxygen inhibition zone” [48], cationic polymerization is oxygen-insensitive [49]. Furthermore, cationic systems typically exhibit much lower volumetric shrinkage compared to acrylates [50]. This dimensional stability stems from the ring-opening mechanism of epoxide groups, which occupies more volume upon opening than the double-bond conversion characteristic of acrylates, thereby mitigating the shrinkage stress that can distort printed appliances.

Despite these advantages, pure cationic systems suffer from significantly slower photoreaction rates compared to radical systems. To address this, researchers have developed acrylic–epoxy hybrid resins (e.g., 3D-1M resin, Okamoto Chemicals) that combine the benefits of both chemistries [51]. In these hybrid systems, the acrylic monomers provide high photosensitivity and rapid initial mechanical strength to stabilize the shape during printing, while the epoxy components contribute to tensile strength and toughness through a secondary curing mechanism. This combination allows for the creation of interpenetrating polymer networks (IPNs) that aim to balance printability with the mechanical demands of orthodontic therapy. Furthermore, these hybrid formulations can be designed using low-toxicity, water-soluble monomers to eliminate the risks associated with skin reactivity and carcinogenicity often found in traditional resins [51].

However, the widespread adoption of these hybrid materials is currently hindered by significant processing and mechanical limitations [51,52,53]. Cationic systems are highly sensitive to humidity, as water acts as a chain transfer agent that can terminate the reaction. Additionally, specific water-soluble epoxy hybrid resins have been found to require approximately five times the energy of general DLP materials to cure effectively, necessitating high-energy printers or longer processing times to ensure polymerization. Moreover, while these materials demonstrate excellent biocompatibility, prototype aligners fabricated from epoxy hybrids have exhibited low fracture toughness, particularly in the middle sections, suggesting that their fracture toughness requires further optimization before they can reliably compete with standard polyurethane-based aligners.

### 2.5. DPA Resins Approved by FDA

Currently, six dental clear aligner resins have received FDA Class II 510(k) clearance for the treatment of tooth malocclusion. Their key information is listed in Table 1. Additionally, Senertek’s directly printed aligner resin, marketed as Clear-A (or Clear-A V2), is CE-certified (Class IIa) for medical use in Europe, but it currently lacks FDA approval.

An analysis of these cleared products reveals how manufacturers have successfully navigated the “Trade-off Triangle” discussed in Section 2.3. Rather than relying on a single monomer family, the majority of these commercial formulations hybridize polyurethane-based systems with methacrylate or acrylate-based networks (e.g., Dreve’s urethanacrylate resin or Graphy’s polyurethane/methacrylate blends). Within this commercial landscape, aliphatic urethane methacrylates currently represent the “gold standard” for DPA resins due to their superior combination of UV stability, toughness, and mechanical performance. This specific molecular architecture provides a validated chemical blueprint: the aliphatic structure prevents UV-induced yellowing over time [54], while the urethane groups provide the necessary hydrogen bonding for mechanical toughness, elasticity, and impact resistance without necessitating excessive cross-linking that would result in brittleness [22,55,56,57]. Meanwhile, the methacrylate functional groups ensure the rapid curing speeds required for viable 3D printing workflows while forming a cross-linked network stiff enough to move teeth. By combining the toughness of polyurethanes with the photoreactivity of methacrylates, these approved products provide a robust material foundation intended to meet the rigorous biomechanical demands of orthodontic therapy, although their long-term clinical efficacy remains to be validated by large-scale in vivo studies.

**Table 1:** DPA resins with FDA Class II 510(k) clearance.

Manufacturer	Resin Composition	Mechanical Properties	Shelf Life	510(k) Number	Approval Date
LuxCreo Inc. (LuxCreo) Belmont, CA, USA	Light-cured polyurethane resin	Flexural strength: 23.6 ± 1.9 MPa; Flexural modulus: 1106 ± 13 MPa; Hardness: 21.6 ± 0.4 HD; Stress relaxation: 37.3 ± 0.3%	6 months	K212680	31 May 2022
Graphy Inc. (Tera Harz Clear) Seoul, Republic of Korea	A blend of polyurethane and methacrylate compounds	Ultimate flexural strength: 58.0–75.7 MPa (varies by model); Flexural modulus: 1575–1849 MPa (varies by model)	24 months	K240597	5 March 2024
LuxCreo Inc. (Modified LuxCreo ) Belmont, CA, USA	Light-cured 3D printing resin (material modification of K212680)	Ultimate flexural strength: 34.6 ± 0.8 MPa; Flexural modulus: 1040 ± 40 MPa; Hardness: 63.0 ± 1.0 HD; Stress relaxation: 41.6%	12 months	K250343	8 April 2025
ODS Co., Ltd. (Clear Miracle) Incheon, Republic of Korea	Methacrylate-based resin	Ultimate flexural strength: 99–105 MPa; Flexural modulus: 1629–1924 MPa	24 months	K251616	22 July 2025
Aidite Tech Co., Ltd. Qinhuangdao, China	Acrylate resin oligomers and acrylate monomers	Ultimate flexural strength: Avg 39.7 MPa; Flexural modulus: Avg 877.5 MPa	24 months	K251415	27 August 2025
Dreve Dentamid GmbH (Primeprint) Unna, Germany	Urethanacrylate resin consisting of methacrylate	Described as demonstrating “similar performance” to LuxCreo (K212680)	Not specified	K250739	7 November 2025

Note: The mechanical properties presented in this table were derived from manufacturer-provided 510(k) summaries. The respective manufacturers state that testing was conducted in accordance with ISO 20795-2:2013 (Dentistry — Base polymers — Part 2: Orthodontic base polymers. International Organization for Standardization: Geneva, Switzerland, 2013); however, these values are self-reported and should not be interpreted as the results of independent, head-to-head comparative study.

## 3. Functional Material Engineering: Exploring Advanced Therapeutic Potential

The shift from solid thermoplastic sheets to liquid resin vats opens the door to functional material engineering. By suspending bioactive nanoparticles or dissolving functional monomers into the printing resin, researchers are exploring ways to expand the aligner’s role beyond a purely passive mechanical device [58,59,60,61,62,63]. Currently, the body of literature regarding functionalized DPAs is disproportionately skewed toward antibacterial modifications. In contrast, advanced therapeutic applications such as active enamel remineralization and sustained drug delivery systems remain in the nascent stages of preclinical exploration. Ultimately, it is important to emphasize that the majority of these functionalized materials—across all categories—are currently confined to the in vitro or proof-of-concept stage, requiring extensive in vivo clinical validation. As printing resins advance and manufacturing workflows become more sophisticated, these emerging functionalities are expected to mature with time.

### 3.1. Antibacterial Functionality

The occlusive nature of aligners restricts saliva flow, buffering capacity, and oxygen exchange throughout treatment periods spanning from 12 to 24 months. This creates a warm, moist and anaerobic microenvironment prone to biofilm accumulation, particularly of *Streptococcus mutans* and *Lactobacillus species* [61,64]. Researchers have successfully imparted antibacterial functionality to TCAs through surface coatings utilizing metallic nanoparticles, such as Zinc Oxide (ZnO), silver nanoparticles (AgNPs) and quaternary ammonium-modified gold nanoclusters [65,66,67,68]. More recently, directly printed aligners containing integrated antibacterial agents have advanced. This novel manufacturing approach demonstrates distinct advantages over surface coatings. Specifically, studies indicate that incorporating antibacterial agents—such as chitosan nanoparticles—into the printing resin at concentrations of 3% to 5% significantly reduces bacterial growth without degrading the polymer’s characteristics [61,69]. Crucially, this integration preserves the aligner’s material properties, ensuring that biological safety, mechanical integrity, and clinical fit are maintained [70].

#### 3.1.1. ZnO Nanoparticles

3D-printed shape-memory resins (Graphy Tera Harz TC-85 DAC) integrated with green-synthesized ZnO nanoparticles (ZnO NPs) have demonstrated significant antibacterial activity against both the periodontopathic bacterium *Porphyromonas gingivalis* (*P. gingivalis*) and the cariogenic pathogen *Streptococcus mutans (S. mutans)* [71]. These particles, typically 15–40 nm in size with a crystalline hexagonal structure, are dispersed into the resin matrix before printing. These nanoparticles exert bactericidal effects through the release of *Zn*^2+^ ions and the generation of reactive oxygen species (ROS) upon exposure to light or biological fluids [62]. These ROS induce oxidative stress in bacteria, damaging cell membranes and DNA. The slow release of Zn^2+^ ions disrupts bacterial enzyme systems and metabolic pathways. Interestingly, *P. gingivalis* showed greater sensitivity, with inhibition zones >25 mm compared to >10 mm for *S. mutans* [71]. Importantly, the antibacterial efficacy of ZnO NPs was found to be durable, lasting for up to 7 days in the studies by Anita et al. [65].

#### 3.1.2. Chitosan Nanoparticles

Direct-printed aligners using Formlabs’ Clear Dental LT V2 resin incorporated with chitosan nanoparticles (ChNPs) exhibit robust antibacterial functionality, characterized by significant antibiofilm activity against *S. mutans* [61]. Research indicates that this efficacy is highly durable; the aligners maintain their antimicrobial properties even after undergoing in vivo aging for the standard one-to-two-week wear cycle. This sustained performance is evidenced by a marked reduction in bacterial colony-forming units (CFUs), with a particularly strong inhibitory effect observed against the standard ATCC laboratory strain, while remaining effective against clinical isolates. Clinically, the integration of ChNPs translates into measurable improvements in periodontal health. Patients utilizing ChNP-modified aligners demonstrated significantly lower plaque index and bleeding on probing scores compared to those using conventional, non-modified aligners.

The underlying mechanism of action is that ChNPs can disrupt bacterial cell wall integrity through electrostatic interactions with cell membranes, leading to intracellular leakage [72]. Furthermore, these nanoparticles may inhibit bacterial proliferation by interfering with mRNA transcription and protein translation [73], thereby providing a comprehensive defense against biofilm-related pathologies during orthodontic treatment.

#### 3.1.3. Quaternary Ammonium Compounds

In addition to the encapsulation of physically dispersed antibacterial nanoparticles within DPAs, the copolymerization of modified quaternary ammonium monomers (QAMs) into the resin matrix represents a significant technological advancement. This approach facilitates the creation of a non-leaching, contact-killing antibacterial surface, which is critical for mitigating plaque retention—particularly on clear aligner attachments [74]. Mechanistically, these positively charged groups interact with negatively charged bacterial cell membranes, disrupting electrical stability and leading to osmotic rupture and cell lysis [75]. Incorporating QAMs like dimethylaminododecyl methacrylate (DMADDM) into orthodontic resins has been shown to reduce *S. mutans* biofilm metabolic activity and lactic acid production by up to 90%, while achieving a substantial 4-log reduction in CFUs [58].

#### 3.1.4. Protein Repellents

Preventing biofilm formation on clear aligners using protein repellents is another antibacterial strategy. This approach simply prevents bacterial colonization on the plastic device without exerting biocidal effects and therefore has minimal impact on the healthy oral microbiome [76,77,78]. This strategy modifies the DPA resin matrix by incorporating zwitterionic materials to create a “non-fouling” interface. Zwitterionic polymers—such as 2-methacryloyloxyethyl phosphorylcholine (MPC) and poly(sulfobetaine methacrylate) (polySBMA)—function by mimicking the phospholipid polar groups found in cell membrane lipid bilayers [79,80]. These materials contain both cationic and anionic functional groups that strongly interact with water molecules. This interaction generates a dense hydration layer of free water around the aligner surface, creating a physical and energetic barrier that effectively repels salivary proteins. Since protein adsorption is the critical initial step that facilitates bacterial anchorage, this hydrophilic barrier disrupts the local microbial balance before biofilm can become established.

Recent research demonstrates that incorporating MPC into photocurable clear aligner resins (such as Tera Harz TC-85) significantly enhances their anti-biofouling capabilities [79]. Experimental data indicates that adding 1% to 3% MPC by weight can reduce the adsorption of proteins, such as bovine serum albumin, to approximately one-third to one-quarter of the levels seen in unmodified resins. This reduction in protein conditioning directly correlates with a decrease in bacterial proliferation; for instance, aligners with 3% MPC showed a 40% reduction in *S. mutans* CFUs compared to controls. This mechanism offers a durable preventive approach to maintain gingival health during orthodontic treatment.

### 3.2. Enamel Mineralization & Caries Prevention

The unique microenvironment created by clear aligners facilitates the adhesion of cariogenic pathogens, particularly *S. mutans* and *Lactobacillus* species. Their colonization contributes to a high incidence of white spot lesions and caries among orthodontic patients. Functional material engineering is required to inhibit bacterial growth and remineralize tooth structure.

To actively reverse demineralization, fillers such as nano-amorphous calcium phosphate (NACP) are being encapsulated into DPA resins to function as ion reservoirs [58,81]. These materials utilize a pH-responsive mechanism: the calcium phosphate fillers dissolve “on demand” to release stored calcium (Ca^2+^) and phosphate (PO_4_^3−^) ions in acidic cariogenic environments [59]. This release supersaturates the local environment, promoting the formation of hydroxyapatite on the enamel surface specifically when the pH drops to critical levels (pH 5.5) [59]. Bioactive glass (BAG) (e.g., Biomin C) is another effective remineralization agent which can be encapsulated into the DPA resins. Alamri et al. proved that the incorporating BAG into orthodontic bonding resins demonstrated a significantly superior remineralization effect compared to traditional non-BAG resins [82]. Moreover, their findings indicated a proportional relationship between filler concentration and the material’s anti-demineralization efficacy.

### 3.3. Drug Delivery Systems

Recent advancements in orthodontic materials have explored functionalizing TCAs with bioactive coatings. Huang et al. have successfully applied cellulose nanofiber coatings loaded with curcumin on PETG material to achieve sustained release for antibacterial and anti-inflammatory effects [83]. However, a significant limitation of this surface-coating approach is its restricted drug-loading capacity. To preserve the aligner’s dimensional fidelity and ensure a precise fit over the patient’s dentition, such coatings must typically remain under 5 µm in thickness [83]. This constraint severely limits the total amount of therapeutic agents that can be delivered.

In contrast, DPAs offer a superior platform for drug delivery by utilizing the bulk material rather than just the surface. Additive manufacturing technology allows for the incorporation of therapeutic agents directly into the printing matrix, effectively creating a monolithic sustained-release system. This approach utilizes the entire volume of the aligner—typically 0.75 mm in thickness—rather than a micron-scale superficial layer. Consequently, this volumetric loading capability can exponentially increase the quantity of the drug encapsulated, enabling more potent therapeutic effects and prolonged release profiles that are difficult to achieve with surface coatings alone.

While literature specifically documenting drug-loaded resins for direct 3D-printed aligners remains limited, the feasibility of this additive manufacturing approach has been demonstrated in orthodontic retainers. Research has successfully developed personalized retainers capable of the sustained release of clonidine hydrochloride (CH) [84]. In this method, the drug is homogeneously mixed with a polymer matrix—a blend of polyethylene glycol (PEG), polylactic acid (PLA), and polycaprolactone (PCL)—and hot-melt extruded to form a printable filament for orthodontic retainer fabrication. This proof-of-concept highlights the potential for additive manufacturing to produce customized devices that function simultaneously as mechanical appliances and pharmacological reservoirs.

Looking beyond passive drug elution, future theoretical models of DPAs might explore the integration of sensing and monitoring components within the polymer matrix. If successfully translated into clinical practice, such long-term innovations could theoretically aid in managing periodontal health or monitoring patient compliance, representing a potential future avenue for personalized orthodontic treatment.

## 4. The Critical Role of Post-Processing

In the fabrication of DPAs, the initial photopolymerization by the 3D printer represents only the first stage of the process. The material’s final properties—including biocompatibility, mechanical strength, and color stability—are critically dependent on subsequent post-processing steps. Inadequate post-processing is a primary cause of mechanical failure and cytotoxicity in printed appliances [85,86].

### 4.1. Cleaning and Washing

After printing, aligners are covered in a layer of uncured toxic liquid resin, which poses significant biological and physical risks. The thorough elimination of these unpolymerized monomers by cleaning and washing processes is a critical prerequisite for achieving biocompatibility and ensuring the dimensional accuracy of the final appliance [85].

Solvent-Based Cleaning Protocols: Isopropyl alcohol (IPA) remains the industry-standard solvent for washing 3D-printed dental appliances [87]. However, its application in clear aligner fabrication is associated with notable drawbacks, including high volatility, flammability, and potential respiratory hazards [88]. Furthermore, prolonged exposure to IPA can compromise the structural integrity of the polymer network. Research indicates that IPA can induce polymer swelling rather than simple dissolution, leading to surface micro-cracks and a reduction in flexural strength [89]. Optical properties are also adversely affected; studies comparing cleaning methods for Tera Harz TC-85 resin demonstrated that IPA-washed samples exhibited significantly lower translucency and a “hazy” appearance compared to those cleaned via centrifugation, attributed to solvent-induced surface degradation [90]. Alternative solvents such as tripropylene glycol monomethyl ether (TPM) have been explored due to their lower volatility and reduced flammability [91]. While TPM may offer a safer working environment, it requires longer washing durations and incurs higher costs. Furthermore, studies suggest that flexural modulus values remain consistent between IPA and TPM groups, indicating that solvent interaction still impacts mechanical properties [87]. It is important to note that a critical balance must be maintained when using any chemical solvent; while extending washing duration can improve cell viability by minimizing residual monomers, excessive exposure to solvents can lead to the extraction of polymer components and a subsequent decline in mechanical properties such as flexural modulus [91].

Centrifugal Washing: To mitigate the deleterious effects of chemical solvents, automated centrifugal washing has emerged as a superior, non-chemical alternative [92]. This method utilizes high-speed rotation (typically 500 to 1000 rpm for 3 to 6 min) to mechanically expel excess uncured resin through g-force. Comparative studies indicate that centrifugation is highly effective at removing residual monomers without inducing the hygroscopic expansion or surface erosion associated with organic solvents. Consequently, aligners processed via centrifugation maintain superior optical clarity and surface smoothness compared to those subjected to solvent baths [92]. Furthermore, centrifugation at elevated temperatures (e.g., 55 °C) can further enhance resin removal efficiency by reducing the viscosity of the uncured material.

In conclusion, adherence to validated, material-specific protocols—whether optimizing solvent immersion times or utilizing advanced centrifugal workflows—is essential to balance biological safety and mechanical stability.

### 4.2. UV Post-Curing

Following fabrication, DPAs exist in a “green state”, characterized by incomplete polymerization and suboptimal mechanical strength [93]. Consequently, post-curing is a mandatory step to maximize the degree of conversion (DC)—defined as the percentage of monomeric double bonds converted into polymeric single bonds—and to stabilize the aligner’s physicochemical properties. To mitigate oxygen inhibition of the polymerization chain reaction, post-curing in an inert (oxygen-deprived) environment is increasingly advocated [94]. Research demonstrates that utilizing a nitrogen-saturated chamber prevents the formation of an oxygen inhibition layer, thereby significantly enhancing the final conversion rate, surface hardness, and cytocompatibility of the aligners compared to those processed in ambient air [95].

### 4.3. Thermal Post-Processing & Double Network Structures

Thermal post-processing serves as a critical adjunct to UV curing, functioning to activate “dual-cure” mechanisms, relieve anisotropic residual stresses inherent to layer-wise fabrication, and facilitate the formation of double-network structures that enhance fracture toughness [96,97,98].

Dual-Curing Mechanisms: Advanced resin formulations for DPAs often employ a “dual-cure” strategy, leveraging thermal energy to complement photochemical initiation. Research on urethane dimethacrylate (UDMA) resins indicates that supplementing UV curing with heat treatment (e.g., at 110 °C) can enhance the formation of IPNs at the interface between printed layers [99]. This thermal facilitation promotes the mobility of polymer chains, allowing for further cross-linking of unreacted monomers that are otherwise trapped in the vitrified network, thereby significantly increasing ultimate tensile strength and internal adaptation [96].

Thermal Annealing: Thermal post-processing can relieve internal stress. The layer-by-layer nature of additive manufacturing introduces anisotropic residual stresses [100,101]; thermal treatment, or annealing, helps to homogenize this stress field, potentially reducing the risk of time-dependent deformation, thereby improving dimensional stability [98]. For shape-memory polymers like Tera Harz TC-85, manufacturer protocols mandate immersion in hot water (e.g., 80 °C for 2 min) as a final processing step [102]. This thermal exposure not only aids in the removal of residual surface monomers but is also hypothesized to organize the polymer network, stabilizing the shape memory properties required for consistent orthodontic force delivery [98].

Double Network Formation: Emerging strategies in additive manufacturing leverage thermal triggers to cross-link secondary polymer networks, dramatically enhancing mechanical performance [103,104,105]. For instance, novel “double thermoplastic” systems have been developed where a stiff linear polymer network is formed via UV printing, followed by a thermal treatment (e.g., 90 °C) that triggers the ring-opening polymerization of a second component, such as *α*-lipoic acid [97]. This sequential curing creates a double network structure where the interaction between the stiff and flexible polymer chains via hydrogen bonding allows for tunable mechanical properties. Such thermal activation can transition a material from a brittle state to a highly tough, flexible elastomer, increasing the strain at break by over two orders of magnitude compared to single-network counterparts [97].

## 5. Biomechanical Performance and Clinical Efficacy

### 5.1. Stress Relaxation and Force Delivery

Current DPA resins on the market still have room for improvement. Studies comparing DPA resins (such as Tera Harz TC-85) to high-end thermoplastics (Zendura FLX, Smart Track) reveal distinct behaviors. While multi-layer thermoplastics are engineered to resist relaxation, Tera Harz TC-85 material initially shows higher stress relaxation [106,107,108]. Other findings suggest that while the initial relaxation is rapid, the material can eventually reach a plateau where the residual static force remains constant under specific in vitro conditions [35,109]. This viscoelastic behavior means that while the material is flexible and resistant to brittle fracture, it may struggle to maintain high force levels continuously without the aid of its shape memory properties.

However, the precision in thickness control afforded by DPAs is significantly superior to that of TCAs, as the thermoforming process inherently results in unpredictable material thinning. This manufacturing precision allows for targeted modifications to aligner thickness, enabling fine-tuning of the magnitude of forces and moments generated [110,111]. Clinicians can design specific areas of varying thickness (ranging from 0.25 mm to 1.2 mm) to control the force vectors and magnitudes applied to specific teeth. Increasing the thickness of the print results in a corresponding increase in force production, allowing for targeted biomechanics that can optimize prescribed movements while minimizing side effects on anchorage teeth. This structural control could help offset the material’s susceptibility to stress relaxation by allowing for the design of stiffer, more resilient localized areas where higher forces are required.

Moreover, DPAs may provide a biologically superior force system for tooth movement. Evidence suggests that at intraoral temperatures, DPAs deliver stabilized forces ranging from approximately 0.73 N to 1.69 N—significantly lower than the 4.60 N to 15.30 N range observed in TCAs [112,113]. This lower force magnitude is considered more biologically favorable, as it mitigates the high initial force spikes typically associated with thermoformed plastics.

### 5.2. The “Shape Memory” Controversy

Marketing for DPAs often highlights “shape memory”, claiming the deformed aligner recovers its original printed shape upon exposure to a thermal stimulus, ensuring sustained force delivery and “active” tooth movement [99]. These resins are typically aliphatic polyurethane polymers with methacrylate functionalization. The shape memory effect (SME) relies on a dual-component polymer network: a stable network (hard segments) that determines the permanent, original shape, and a reversible switching network (soft segments) that allows the material to deform into a temporary shape.

The material functions as a temperature-responsive polymer. While some studies report a glass transition temperature (Tg) around 42.3 °C, the heat absorption curve can begin as low as 30.4 °C, allowing the shape memory effect to activate within the oral environment (37 °C) [35,99]. Other studies suggest higher activation temperatures (e.g., 60 °C) are needed for activating the SME and recovering the aligner’s orthodontic force [114]. In laboratory settings, Tera Harz TC-85 specimens that were bent at high temperatures and cooled to retain a temporary shape demonstrated a shape recovery of more than 50% within the first minute of immersion in 37 °C water [35]. Approximately 90% of the deformation is recovered within 10 min, reaching 96% recovery after 60 min [115].

While DPA materials are often marketed as delivering Nickel-Titanium (NiTi)-like forces, distinct mechanical differences remain (Table 2). Despite the shape memory nature of these polymers and the theoretical force plateau discussed previously, a critical paradox defines the DPA shape-memory: geometric recovery does not equate to sustained mechanical force retention. While the material recovers its geometry well (e.g., 96% recovery) [35], its printed polymer network is highly vulnerable to water absorption and plasticization in the intraoral environment. Consequently, it struggles to maintain the mechanical force required to move teeth over time when exposed to constant moisture and body temperature (37 °C). For instance, Avolese et al.’s study indicates that under constant strain, the stress relaxation of 3D-printed aligner made with Tera Harz TC-85 DAC resin is rapid in a 37 °C water bath, with one extreme case showing a decay of up to 100% in force, resulting in a residual force of nearly 0 N in the simulated oral conditions [115].

Furthermore, direct-printed aligners are engineered to activate as temperatures approach Tg, where polymer cross-linking enables the shape memory effect. However, 3D-printed resins—particularly those containing polyurethane—exhibit high susceptibility to hygroscopic expansion, with water absorption levels reaching up to 8.7% after two weeks of immersion [86]. Within the oral environment, this moisture absorption can decrease the Tg and degrade the polymer network [116]. This results in a reduced elastic modulus, which ultimately compromises shape memory performance over the standard 1–2 weeks wear cycle.

**Table 2:** Comparison of shape memory property between DPA resin vs. NiTi material.

Feature	Shape Memory DPA Resin	NiTi Alloy
Material Nature	Cross-linked polymer network (organic)	Metallic crystal (inorganic)
Memory Mechanism	Molecular chain relaxation (utilizing entropy-driven recovery near the Tg)	Phase transformation (relying on a stress-induced martensitic transformation (superelasticity) that is intrinsic to the alloy’s crystal structure)
Primary Stimulus	Temperature (body heat or warm water activation).	Temperature (body heat) or stress (superelasticity).
Stress Relaxation	High. Exhibits significant stress relaxation (force loss) within the first few hours of loading.	Low. Maintains active force levels for weeks without significant relaxation, making it highly efficient for long-duration activation.
Elastic Modulus (Stiffness)	Low. Approximately 40–50 times lower than NiTi. The material is more easily deformed under load.	High. High stiffness allows it to resist deformation and exert force even under heavy loading conditions.
Resiliency	Moderate/Low. Absorbs energy but dissipates much of it as heat (hysteresis) rather than returning it as active force [21,117].	High. Excellent ability to store and return energy (spring-back) without permanent deformation [118].
Clinical Advantage	Selective thickness; superior fit; constant “gentle” force for improved comfort.	High stiffness and force; ideal for heavy tooth movement.

### 5.3. Clinical Evidence

The transition of DPAs from laboratory testing to clinical application has begun to yield initial quantitative data regarding their efficacy [119,120]. However, it must be explicitly acknowledged that the current body of clinical evidence remains limited. At present, data is predominantly derived from short-term pilot studies and retrospective cohorts, with a notable absence of long-term, large-scale randomized controlled trials. Caution must be exercised to avoid extrapolating laboratory or short-term clinical results to predict long-term clinical performance. While TCAs utilize auxiliary attachments to enhance grip and force delivery, early clinical studies using shape-memory resins (e.g., Tera Harz TC-85) suggest DPAs can achieve predictable outcomes in mild-to-moderate cases with minimal auxiliary use. A prospective observational pilot study reported an overall accuracy of 72.0% for rotational movements, with specific teeth such as maxillary central incisors achieving 84.9% accuracy [120]. Notably, this high degree of rotational control was achieved with rectangular attachments applied to only seven teeth across the entire study sample, suggesting a theoretical potential for reduced reliance on composite attachments for difficult movements. Furthermore, a retrospective cohort study comparing DPAs to TCAs found no statistically significant differences in the accuracy of torque (60% vs. 66%), tip (52% vs. 61%), or rotation (58% vs. 69%), suggesting that in-house printed appliances may perform comparably to established thermoformed protocols in selected mild-to-moderate clinical scenarios [119]. Nevertheless, these preliminary findings must be interpreted with caution until validated by broader and larger clinical trials.

Extrusions and rotations are notoriously difficult for aligners [121]. DPAs allow for printing of varying thicknesses—e.g., thickening the aligner at the gingival margin (e.g., from 0.5 mm to 0.7 mm) to increase stiffness for anchorage or on specific surfaces to optimize force vectors [48]. This geometric freedom potentially improves control over these movements without the need for excessive attachments. Experimental studies confirm that increasing the labial or lingual thickness of the aligner can alter the magnitude of forces and moments generated, reducing unwanted side effects on adjacent anchorage teeth during bodily movements [110]. Despite this geometric freedom, generating pure bodily movement remains biomechanically complex; variations in thickness result in complex force patterns that are difficult to predict, often necessitating the continued use of auxiliaries or overcorrection to ensure clinical success [90].

## 6. Biocompatibility, Microplastics, and Safety

The introduction of any new biomaterial into the oral cavity requires rigorous safety validation. DPAs present unique challenges compared to inert thermoplastic sheets, specifically regarding the elution of uncured monomers and the generation of microplastics (MPs).

### 6.1. The Monomer Elution Risk

Unlike thermoplastics, which are chemically static plastics formed from fully polymerized discs, DPA resins are toxic and allergenic in their liquid state, relying entirely on the printing and post-curing phases to achieve a stable, biocompatible solid [21,48]. Consequently, if the polymerization reaction is not complete, the presence of residual monomers (e.g., UDMA, TEGDMA) and photoinitiators within the polymer network that can leach into the oral environment, and introduce the concern of potential health hazards [122].

Research indicates that the DC is a critical determinant of this risk; Studies conducted by Willi et al. on the widely used Tera Harz TC-85A resin demonstrated a DC of approximately 83%. This incomplete polymerization leaves a margin for residual monomer elution during 1 week immersion tests at 37 °C, quantifiable amounts of UDMA (ranging from 29 to 96 μg/L) were detected in water eluates [123].

Long-term elution studies (e.g., 14 days) in the simulated oral condition are critical, as aligners are worn for extended periods. The elution raises concerns because aligners are replaced every 1 to 2 weeks. This frequent renewal subjects the patient to a cumulative and recurrent exposure to the initial high flux of monomer release that occurs with every new appliance [122], a scenario distinct from permanent dental restorations where elution decreases exponentially over time. While in vitro studies suggest these elution levels remain below cytotoxic thresholds for human gingival fibroblasts [124], UDMA is known to have genotoxic potential and can induce oxidative stress at higher concentrations.

The magnitude of monomer elution risk is critically dependent on the post-processing protocols discussed in Section 4. While an intrinsic risk of elution exists due to the nature of in situ polymerization, it can be effectively mitigated through strict adherence to validated workflows that ensure high DC and surface stability [85]. For instance, when processed according to manufacturer specifications—specifically utilizing a nitrogen-saturated post-curing unit (e.g., Tera Harz Cure) alongside rigorous washing cycles—TC-85 aligners demonstrated high biocompatibility. In fact, experimental assays showed cell viability rates exceeding 100%, indicating no inhibition of cell growth and confirming the safety of the material when properly cured [122]. However, it must be emphasized that current concerns regarding the systemic toxicity of these eluted monomers in orthodontic patients remain largely theoretical. Long-term in vivo clinical studies are currently lacking, and such data is necessary to accurately quantify the biological impact of this cumulative intraoral exposure.

### 6.2. Microplastics

A critical concern distinct to additive manufacturing is the anisotropic, layer-wise microstructure of the printed appliance. Intraoral mechanical loading (due to mastication, bruxism) can induce fatigue at these layer interfaces, potentially causing delamination and the subsequent release of microplastics. Preliminary studies indicate that, due to this structural irregularity, DPAs may generate larger and more abundant microparticulate debris compared to the homogeneous surfaces of conventional extruded thermoplastics [125]. Compared to Invisalign aligners, DPAs also exhibited a greater increase in surface roughness after intraoral use [126]. The majority of these particles fall within the 5–20 μm range [127]. This size is biologically significant; particles <10 μm can theoretically be phagocytosed by macrophages or penetrate gut epithelia, although current evidence suggests most 5–20 μm particles are excreted.

The weight of microplastics separated from DPA samples (Tera Harz TC-85 DAC resin) after mechanical rubbing was 0.004 g per 200 µL, which is four times higher than the 0.001 g per 200 µL released by TCAs (specifically Invisalign SmartTrack) [125]. Particles detached from DPAs were significantly larger and more numerous, covering 32.34% of the microscopic field compared to only 1.07% for TCA samples. TEM analysis confirmed that the average grain size of DPA particles was approximately 1000 times larger than that of TCA particles [125]. While laboratory tests indicate higher microplastic generation, the actual clinical ingestion rates and systemic health impacts of these specific 5–20 μm particles remain theoretical and require future biological investigation.

Higher microplastic release in DPAs can be attributed to several factors:

(a) The layer-by-layer structure of DPAs: The additive manufacturing process (layer-by-layer printing) inadvertently introduces microscopic ridges, pores, and spaces. These irregularities serve as potential niches for structural failure and facilitate the detachment of larger clusters of material under stress or shear forces during chewing, which can cause delamination or the shedding of microscopic step-edges [125,128,129].
(b) Incomplete polymerization: Incomplete polymerization (low DC) fundamentally compromises the material’s structural integrity, making it more susceptible to the mechanical wear that sheds plastic particles [129,130]. Resins with a low DC possess lower flexural strength and hardness. This makes the material “softer” and more prone to chip formation, crack nucleation, and surface fractures when subjected to cyclic loading, such as the forces of swallowing or mastication. Moreover, materials with low DC are more susceptible to hydrolytic degradation in the oral environment. As the polymer network breaks down chemically due to moisture and enzymes, it weakens physically, facilitating the detachment of secondary microplastics. Incomplete UV curing is a known cause of increased surface roughness and porosity. Rougher surfaces have higher friction coefficients and more microscopic “peaks” that can be mechanically sheared off as microplastics during function [131].

The microplastic release is a significant area for future research, necessitating the development of tougher, more abrasion-resistant resins or surface coatings to seal the printed layers and mitigate microplastic ingestion [125].

## 7. Conclusions & Future Outlook

The transition from conventional TCAs to DPAs signifies a fundamental shift in orthodontic manufacturing, effectively addressing the geometric and manufacturing constraints inherent to the thermoforming process [102,132]. Unlike the subtractive nature of thermoforming—which is characterized by unpredictable material thinning and substantial model waste—direct additive manufacturing enables precise, voxel-level control over appliance geometry. This evolution has yielded appliances with superior dimensional stability, enhanced fit accuracy, and the unique capability for variable thickness profiles [10,11,17,18,89]. Consequently, DPAs optimize force delivery systems while reducing the solid waste associated with single-use resin models. However, the overall sustainability of DPAs must be interpreted cautiously; comprehensive, quantitative life cycle assessments are still required to fully evaluate their ecological impact.

Despite significant advancements, material optimization remains an ongoing challenge. Current resin formulations, particularly those based on aliphatic urethane methacrylates, have demonstrated preliminary clinical efficacy comparable to premium thermoplastic clear aligners in pilot studies [119,120]. Nevertheless, there is currently a notable absence of large-scale, long-term randomized controlled trials. Consequently, these initial findings must be interpreted with caution and should not be extrapolated to predict long-term clinical performance. Contemporary shape-memory resins, exemplified by materials like Tera Harz TC-85, demonstrate the capacity to recover their original geometry under thermal stimuli in laboratory settings, theoretically exhibiting favorable viscoelastic properties that may help maintain force levels closer to the biological ideal. However, as discussed regarding the shape-memory paradox, a hurdle remains in translating this geometric recovery into long-term force retention. When exposed to simulated intraoral conditions (i.e., moisture and 37 °C), most current DPA materials remain highly susceptible to rapid stress relaxation, often losing force faster than premium thermoplastic materials. Therefore, room for improvement exists in balancing the “Trade-off Triangle” of transparency, toughness, and stress relaxation. The next phase of material science must bridge the remaining biomechanical gap between printed networks and established thermoplastics. In this regard, the integration of artificial intelligence is anticipated to aid in the next generation of resin formulation, computationally optimizing these complex material trade-offs to meet patient-specific needs [133,134,135].

Looking forward, preclinical research in functional material engineering explores the potential to transform aligners from static devices into bioactive and stimuli-responsive systems. By leveraging functional materials and sophisticated design strategies, theoretical models suggest that future DPAs could autonomously stage complex movements within a single aligner [8,53,136]. If successfully translated into clinical practice, this capability would theoretically reduce the total number of appliances required for treatment, thereby exploring new avenues for clinical efficiency and resource utilization. Alongside these functional innovations, addressing the environmental burden of clear aligner plastic waste [137,138] through the development of bio-based and biodegradable resins [139,140,141] represents another necessary avenue for future material research.

Ultimately, the successful widespread adoption of DPAs has the potential to drive the maturation of orthodontics into a fully digital discipline. If it successfully closes the loop between virtual planning and physical fabrication, this technology will provide the foundational framework to better align the precision of the therapeutic appliance with the sophistication of the digital diagnosis, pending further long-term clinical validation.

## Data Availability

Data sharing is not applicable to this article as no new primary data were created or analyzed in this study. All synthesized data, including the product specifications in Table 1, were sourced from publicly available repositories such as the FDA 510(k) database.
